# Physicochemical Characterization and Biological Activities of the Triterpenic Mixture α,β-Amyrenone

**DOI:** 10.3390/molecules22020298

**Published:** 2017-02-16

**Authors:** Rosilene G. S. Ferreira, Walter F. Silva Júnior, Valdir F. Veiga Junior, Ádley A. N. Lima, Emerson S. Lima

**Affiliations:** 1 Higher Normal School, University of the State of Amazonas, Av. Djalma Batista 69050-010, Brazil; rgsilva@uea.edu.br; 2 Laboratory of Biological Activity, Faculty of Pharmaceutical Sciences, Federal University of Amazonas, Av. General Rodrigo Otávio, 69077-000-Manaus-AM, Brazil; 3 Department of Pharmacy, Federal University of Rio Grande do Norte, Av. Coronel Gustavo Cordeiro de Farias s/n, 59012-570-Natal-RN, Brazil; walterjuniornt@hotmail.com (W.F.S.J.); adleyantonini@yahoo.com.br (Á.A.N.L.); 4 Laboratory of Chemistry of Amazonian Biomolecules, Department of Chemistry, Federal University of Amazonas, Av. General Rodrigo Otávio, Manaus, AM 69077-000, Brazil; valdir.veiga@gmail.com

**Keywords:** enzymatic activity, triterpenes, *Protium*, amyrin, amyrenone

## Abstract

α-Amyrenone and β-amyrenone are triterpenoid isomers that occur naturally in very low concentrations in several oleoresins from Brazilian Amazon species of *Protium* (Burseraceae). This mixture can also be synthesized by oxidation of α,β-amyrins, obtained as major compounds from the same oleoresins. Using a very simple, high yield procedure, and using a readily commercially available mixture of α,β-amyrins as substrate, the binary compound α,β-amyrenone was synthesized and submitted to physico-chemical characterization using different techniques such as high-performance liquid chromatography, nuclear magnetic resonance (^1^H and ^13^C), mass spectrometry, scanning electron microscopy, differential scanning calorimetry, thermogravimetry and derivative thermogravimetry, and Fourier transform infrared spectroscopy (FTIR). Biological effects were also evaluated by studying the inhibition of enzymes involved in the carbohydrate and lipid absorption process, such as α-amylase, α-glucosidase, lipase, and their inhibitory concentration values of 50% of activity (IC_50_) were also determined. α,β-Amyrenone significantly inhibited α-glucosidase (96.5% ± 0.52%) at a concentration of 1.6 g/mL. α,β-Amyrenone, at a concentration of 100 µg/mL, showed an inhibition rate on lipase with an IC_50_ value of 82.99% ± 1.51%. The substances have thus shown in vitro inhibitory effects on the enzymes lipase, α-glucosidase, and α-amylase. These findings demonstrate the potential of α,β-amyrenone for the development of drugs in the treatment of chronic metabolic diseases.

## 1. Introduction

The binary compound α,β-amyrenone is a triterpenic derivative of the ursane and oleanane series commonly described in isolation studies from Burseraceae family species such as *Protium heptaphyllum*, *P. opacum* var. *opacum,* and *P. giganteum*; *Trattinnickia glaziovii* and *T. peruviana* [1,2]. According to some authors, there are other sources in which α,β-amyrenone was identified such as: *Cissus quadrangulares*, *Tridax procumbens*, *Camellia sinensis* var. *sinensis*; *Beilschmiedia alloiophylla*, *Pistacia lentiscus* (resin, found in archaeological remains); *Beilschmiedia* sp.; *Anacolosa pervilleana*; *Ficus microcarpa*; *Ficus pandurata* and *Cyclocarpa paliurus* [3,4,5,6,7,8].

Previous studies using α,β-amyrenone have demonstrated its pharmacological potential. A study involving the oral administration of α-β-amyrenone mixture to mice demonstrated its ability to reduce mechanical hypersensitivity and carrageenan-induced paw edema, and interference with neutrophils migration was studied as well. [9]. Our group has also reported that the same mixture inhibited the production of nitric oxide and Interleukin 6 (IL-6) and induced the production of IL-10 in murine macrophages J774 stimulated by Lipopolysaccharide (LPS), in addition to the inhibition of cyclo-oxygenase-2 (COX-2) expression. In the same study, α-β-amyrenone inhibited ear and paw edema induced by carrageenan [10].

Despite this, little is known about the physico-chemical characteristics of this molecule, which might allow, for instance, the preparation of pharmaceutical formulations. Herein, we aimed to characterize α-β-amyrenone chemically and physico-chemically and to verify its in vitro effects in relation to the inhibition of enzymes involved in the carbohydrate and lipid absorption process, such as α-amylase, α-glucosidase and lipase, that may be useful for the study of the mechanism of action in metabolic diseases, among others.

## 2. Results and Discussion

**Figure 1:**
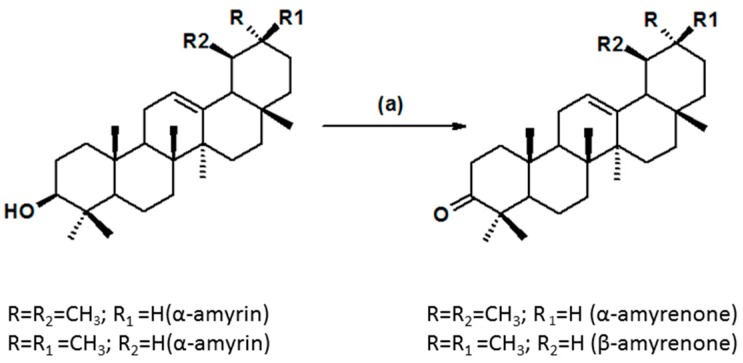
The synthesis of oxidized derivatives of a mixture of α-amyrin (R_1_ = H, R_2_ = CH_3_) and β-amyrin (R_1_ = CH_3_, R_2_ = H). (a) Pyridinium-chlorochromate (CCP), CH_2_Cl_2_.

The mixture of α,β-amyrenone was obtained by oxidation of α,β-amyrin isolated from *Protium* Amazonian oleoresins. The reaction yield based on 1.0 g of α,β-amyrin was about 70% of α,β-amyrenone with approximately 0.9966 of purity, according to [11]. The procedure was repeated nine times, to obtain the larger amount of 6.9 g of α and β-amyrenone isomers to perform all the assays of this study (Figure 1).

**Figure 2:**
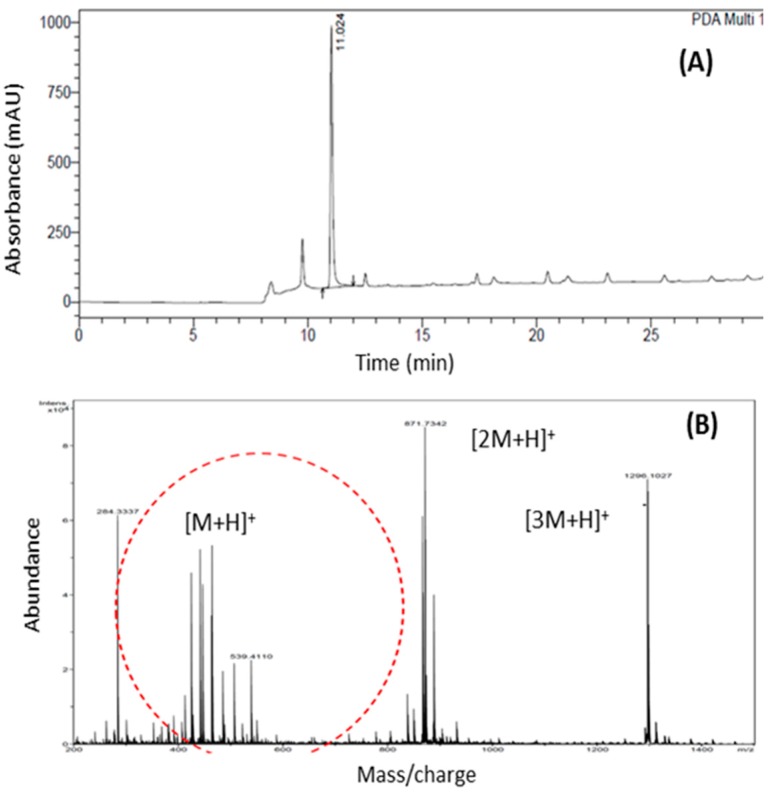
HPLC: (**A**) peak hold of α,β-amyrenone mixture; (**B**) Peak purity index of 0.996605 of α and β-amyrenone.

All oxidation processes were monitored by Thin-layer chromatography (TLC) using hexane/ethyl acetate as eluents (9.5/0.5) and visualized with sulfuric vanillin. Assessment of the purity of amyrenone and confirmation of the synthesis were performed by HPLC analysis (Figure 2A). Amyrenone was analyzed by electrospray ionization mass spectrometry showing a molecular ion peak [M + H]^+^ at *m*/*z* 465, consistent with the molecular formula C_30_H_47_O, according to the mass spectrum (Figure 2B).

The efficiency of the oxidation of the C3 carbinolic carbon was confirmed by the NMR technique. The characteristic signs were observed both by the ^1^H shift analysis for the absence of the carbonyl carbons in δ = 3.23 ppm and detection of displacements in δ = 2.25 ppm, as well as a similar ^13^C-NMR displacement profile, demonstrating efficiency of synthesis methodology.

Infrared spectroscopy provides information about bonds whose dipole moment changes during vibration, which enables analysis of short-range order and is a valuable technique for the identification of the functional groups of the analyzed structures [12]. In the spectrum obtained from α,β-amyrenone, there is a band related to stretching and bending vibrations of the C(=O)–C bond between 1100–1230 cm^−1^. The stretch vibration of C=O bond appeared intense in 1705 cm^−1^. A band of increased intensity was observed between 2850 and 2950 cm^−1^ and is associated to vibrations of axial deformation of the hydrogen atoms bonded to carbon, being a characteristic of C-H bonds in cyclic chains. Therefore, great similarity was observed in its structure, whereas the major part is composed of cyclic chains. Two intense bands at 1450 cm^−1^ and 1375 cm^−1^ were observed and are related to the bending vibrations of the C-H bond of methyl and methylene groups, respectively [13,14].

### 2.1. Physicochemical Characterization of α,β-Amyrenone

**Figure 3:**
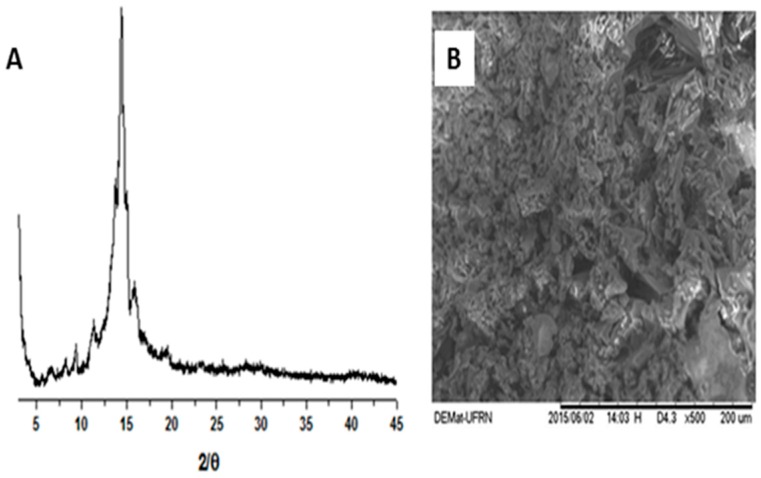
X-ray diffraction pattern of α,β-amyrenone (**A**); and surface morphological appearance of α,β-amyrenone (**B**).

The shapes and surface characteristics of α,β-amyrenone are shown in Figure 3B. The micrographs revealed porous particles with arrays of crystalline systems with irregular shapes and sizes. The crystallinity of the sample was confirmed by X-ray diffraction (XRD) analysis as shown in Figure 3A.

According to the concept of crystalline powders, these solids are characterized by having three-dimensional structures that are able to diffract X-rays and exhibit a well-defined melting point [15]. It was possible to observe the diffraction peaks that characterize the substance as a crystal lattice, in which it was observed intense and superimposed crystalline reflections around 13° to 15°. These characteristics possibly result in its low aqueous solubility observed in previous qualitative experiments, that showed that 1 mg of α,β-amyrenone was added in increasing amounts of water, starting from 1 mL up to 1000 mL. It was verified that even with addition of water, no solubilization of α,β-amyrenone was not observed, characterizing it as a water-insoluble substance. Taking into account the crystalline state of α,β-amyrenone, it can be determined that this characteristic directly influences its dissolution and, consequently, its bioavailability, due to the fact that its crystalline arrangement decreases the contact surface of the drug and interferes with the penetration of the solvent, which may reduce its solubility [16].

**Figure 4:**
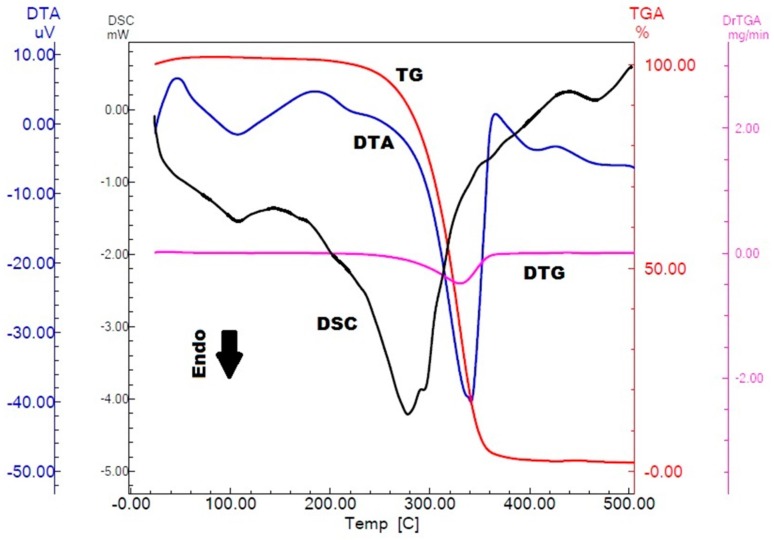
Differential thermal analysis (DTA), differential scanning calorimetry (DSC) and thermogravimetry (TG)/derivative thermogravimetry (DTG) curves for α,β-amyrenone in heating rate of 10.0 °C·min^−1^.

The thermogravimetry (TG)/derivative thermogravimetry (DTG), differential thermal analysis (DTA) and differential scanning calorimetry (DSC) curves of α,β-amyrenone are shown in Figure 4. The TG curve shows a single step of mass loss of approximately 95%. The DTA curve shows two endothermic events represented by descending peaks. The physical event that does not involve loss of mass is demonstrated only in the DTA curve. Thus, the analysis using the association of both thermoanalytical techniques is important for the characterization of the sample studied.

Analyzing the curves shown in Figure 4, it was observed that in the TG curve, α,β-amyrenone is thermally stable up to a temperature of 235 °C. The decomposition of α,β-amyrenone occurs between 235 °C and 265 °C, which is visualized as a single step in the TG curve. This event is indicated by the peak in the DTG at 327 °C, point at which the mass changes more rapidly. The area of the DTG peak is directly proportional to the mass variation of the sample, and the value found for α,β-amyrenone was 2.720 mg, corresponding to 95% of mass loss.

Between 78 °C and 115 °C occurs an endothermic event that corresponds to the α,β-amyrenone melting point, at which there is no evidence of mass loss by the TG and indicates that the physical event of change from solid to liquid state occurs, confirming the natural crystallinity of α,β-amyrenone. After the fusion event, there is a second endothermic event observed in the DTA, between 295 °C and 363 °C, which corresponds to loss of mass of α,β-amyrenone, and it was confirmed by TG. DSC curves were shown to be similar to the DTA curves. The first endothermic event in DSC represents the melting range of α,β-amyrenone. The event has an onset temperature of 81 °C, an endset of 122 °C, and a maximum peak temperature of 107 °C. The enthalpy value generated in the event was −24 J/g. The second endothermic event is related to the mass loss of α,β-amyrenone, which had an onset temperature of 242 °C, an endset of 320 °C, and a maximum peak temperature of 278 °C. The enthalpy generated in the event was −392 J/g.

### 2.2. Biological Assays

Bioassays allowed the determination of the inhibitory activity of α,β-amyrenone in relation to three important enzymes in metabolic and pathological processes. The inhibitory assays for lipase, α-amylase and α-glucosidase in vitro were carried out using the binary mixture α,β-amyrenone.

**Figure 5:**
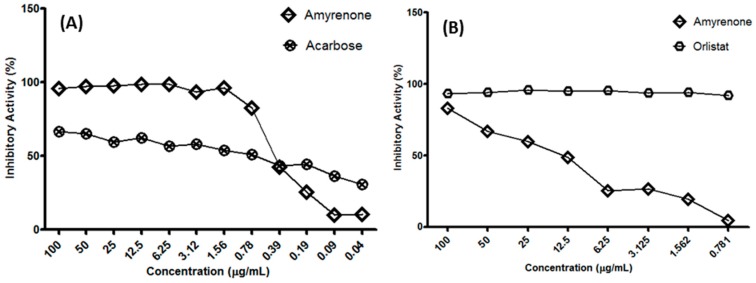
(**A**) Inhibitory Activity (%) of isomers of α,β-amyrenone on α-glucosidase enzyme. The data analyzed in *t* test demonstrated value of 0.0002; (**B**) Inhibitory Activity (%) of α,β-amyrenone on pancreatic lipase enzyme and comparison with the standard orlistat.

The results of the α-glucosidase inhibition activity, in comparison with the standard acarbose, demonstrated that a concentration of 1.6 µg/mL of the sample under study reached a 96.59% inhibition rate (standard deviation ± 0.52), while the standard reached an inhibition rate of 51.5% at a concentration of 60 μg/mL. Thus, α-β-amyrenone demonstrated greater activity (Figure 5).

For tests conducted with enzyme of mammalian intestine rat the inhibition was 35.6% ± 0.46% in a concentration of 100 µg/mL. As the inhibition rate was lower than 50%, the curve for this test was not performed. Using a yeast (*Saccharomyces cerevisiae*) α-glucosidase, a percentage of inhibition greater than 90% and an IC_50_ (0.392 μg/mL) nearer to the standard (0.172 µg/mL) was observed, as also shown in the mixture of α,β-amyrenone isolated from *Protium* *heptaphyllum* [11]. This may be due to the antioxidant effects of this triterpene or, as reported, to the oleanoic acid to reduce glucose levels [17]. Studies have shown that triterpenes, especially pentacyclic, may have insulin-sensitizing properties, that also can be a path to explain the hypoglycemic effect previuosly observed in α,β-amyrenone [18].

Our data demonstrate that α,β-amyrenone mixture showed a significant percentage of inhibition (%) to lipase. By presenting significant inhibitory capacity greater than 80%, it was determined the inhibitory concentration for 50% of enzyme activity (IC_50_) calculated by the Origin program (Origin Lab Corp, Northampton, MA, USA), through a non-linear progression analysis. Lipase inhibitory analysis resulted in an IC_50_ value of 1.193 ± 0.41 µg/mL. However, if compared with the standard orlistat, we observed that the mixture was not effective in inhibiting lipase considering that in a concentration of 3.125 µg/mL the standard inhibits 78.08% of the enzyme activity, and the mixture of α and β-amyrenone shows an inhibition index lower than 50%.The inhibitory activity obtained for α-amylase enzyme was approximately 25%. Since the inhibition value was below 50%, at a concentration of 100 µg/mL, an IC_50_ curve was not produced.

## 3. Materials and Methods

### 3.1. General Information

*Biological material*: 2 kg of white *Protium* spp. oleoresin were purchased in the market of Coari, Amazonas State, in Brazil. The samples were cleaned of impurities, such as mineral residues (sand and others). After the cleaning process, the samples were ground in a porcelain crucible, identified, and stored in the refrigerator until the extraction procedure.

*Amyrin extraction*: Amyrin was isolated from the commercial oleoresin and its isolation was performed by normal phase column chromatography. A crushed sample (3 g) of was used directly in the column (3.5 of internal diameter and 85 cm of height). The sample was subjected to a chromatographic filtration on silica gel 60 (0.063–0.200 mm, 70–230 mesh), and hexane, ethyl acetate (EtOAc) and methanol were the eluents used in increasing order of polarity. In analysis by thin-layer chromatography (TLC), fraction F4 showed spots with *R*_f_ features of the isomers α and β-amyrin, compared with a commercial standard. Therefore, washing was performed with the solvents acetone and methanol (five times in each solvent) in an ultrasound bath for 10–15 min. At each washing, the samples were filtered through an analytical filter. After the process, TLC was carried out. The obtained α and β-amyrins were used in the oxidation process.

*Oxidation:* For the oxidation process, pyridinium-chlorochromate (PCC, 700 mg) were added to a solution containing α and β-amyrin (1.0 g) in dichloromethane (30 mL) [11]. The solution was kept under stirring at room temperature until all the substrate had been consumed. The oxidation process was monitored by TLC at times 30, 40, 60, 24 and 120 min and 30 h to observe the completion of the synthesis. After the oxidation reaction was over, ethyl ether was added to the mixture, resulting in formation of a dark precipitate, which was washed several times with the same solvent. The ether solution was filtered over silica gel, and the eluate was evaporated to generate the mixture of triterpenes studied here. At the end of the process, TLC as carried out using the mixture hexane/EtOAc (95:5) as eluent and silica gel as the stationary phase.

*Fourier Transform Infrared (FTIR) spectroscopy*: IR analysis of α,β-amyrenone was performed using an IR Prestige-21 instrument (Shimadzu Corporation, Kyoto, Japan). Tablets of the substance in analysis were prepared with potassium bromide (KBr) in appropriate proportions, by submitting the mixture to 10 tonnes of pressure in a hydraulic press. The analysis was performed in the region of 400 to 4000 cm^−1^ with 15 scans and a resolution of 4 cm^−1^.

*NMR*: For this test the equipment used was a 300 MHz Model, 300 Fourier Transform Nuclear Magnetic Resonance instrument (Bruker Corporation, Billerica, MA, USA), 550 μL of deuterated chloroform as solvent and 5 mm NMR tubes.

*Mass Spectrometry (MS)*: Electrospray ionization quadrupole time-of-flight mass spectrometry (ESI-QTOF-MS) measurements were carried out in SYNAPT HDMS instrument (Waters, Manchester, UK). Samples were infused directly into the instrument’s ESI source at a flow rate of 8 µL/min. Typical acquisition conditions were capillary voltage 3 kV, sampling cone voltage 30 V, source temperature 100 °C, desolvation temperature 200 °C, cone gas flow 30 L·h^−1^, and desolvation gas flow 900 L·h^−1^. ESI (+) mass spectra (full scans) and fragment ion spectra for quadrupole-isolated ions (QTOFMS/MS) were acquired in reflectron V-mode at a scan rate of 1 Hz. For fragment ion experiments, the desired ion was isolated in the mass-resolving quadrupole, and the collision energy of the trap increased cell was sufficient until the fragmentation was observed. Argon was used as the collision gas. Prior to all analyzes, the instrument was calibrated with phosphoric acid oligomers (00:05 H_3_PO_4_% in H_2_O/MeCN 50:50) between *m/z* 90 and 2000. Data acquired were processed with the MassLynx v.4.1 software.

*HPLC*: Acetonitrile and water were used as the mobile phase, applying the method described by [19], with a running time of 30 min and flow rate of 0.8 mL/min. The sample (10 µL) was injected for each run and a 5 µm C18 (2) 100 Å 250 × 4.6 mm Luna column (Phenomenex, Torrance, CA, USA) was used. The standard α and β-amyrin was from Sigma-Aldrich (St. Louis, MO, USA) and the standard mixture of α and β-amyrenone was isolated from a sample of resin.

### 3.2. Physico-Chemical Characterization

*Scanning electron microscopy (SEM) and X-ray diffraction*: the morphology of ABAM particles was assessed by using scanning electronic microscopy (SEM) images recorded on a Tabletop Microscope model TM-3000 (Hitachi Ltd., Tokyo, Japan) using 15 kV. The X-ray diffraction analysis was completed on a D2 Phaser instrument (Bruker Corporation, Billerica, MA, USA) using CuKα (λ = 1.54 Å) radiation with a Ni filter at a pitch of 0.02°, with 10 mA of current, at 30 kV, using a Lynxeye detector (Bruker Corporation, Billerica, MA, USA).

*Thermal behavior*: differential scanning calorimetry (DSC) measurements were carried out on a DSC Q20 cell (TA Instruments, New Castle, DE, USA) using a hermetically sealed aluminum crucible. About 4 mg of sample were used for all the experiments, under a dynamic nitrogen atmosphere (50 mL/min), at a heating rate of 10 °C/min in a temperature range of 25 to 500 °C. The temperature and heat flow of the DSC instrument were calibrated with indium (melting point = 157.5 °C and ∆H = 26.7 J/g). Thermogravimetry (TG) curves were obtained on a TGA-60H instrument (Shimadzu Corporation, Kyoto, Japan) using the similar conditions as for the DSC experiments, at an interval of 35 to 900 °C. The thermoanalytical data were analyzed by universal TA 2000 software.

### 3.3. Enzymatic Inhibition Assays

*α-Glucosidase inhibition assay*: The inhibitory activity of α-glucosidase was based on [20]. This experiment involved enzyme from rat intestinal acetone powder (Sigma-Aldrich, I1630) and from the yeast *Saccharomyces cerevisiae* (Sigma-Aldrich, G0660). Briefly, after the dilution of the substance, the reaction was performed on microplate with the prepared enzyme solution (10 U/mL to G0660 and 3 mg/mL to I1630). Then, there were obtained the values of the absorbances at 405 nm. With the addition of color reagent, readings were carried out every 5 min until the time of 30 min. The values obtained on microplate reader were converted in percentage of inhibition:

% Inhibition = 100 − (final abs − sample initial abs/final abs − initial abs of control) × 100.

*Lipase inhibition assay*: The inhibition of lipase activity was determined after [21], with modifications. The isolated substance was diluted in dimethyl sulfoxide (DMSO), with serial dilutions since 1 mg/mL. 30 µL of inhibitor were added into the microplate at concentration of 1 mg/mL (substance, diluent, control, DMSO, standard). Thereafter, we added 250 µL of the enzyme solution (Sigma-Aldrich L-3126, 1 mg/mL). The sample was incubated for 5 min at 37 °C. After adding the color reagent PNP and incubating, the first reading of testing was performed in ELISA reader at absorbance of 405 nm. The mixture was homogeneized, in order to avoid the formation of 2 phases, incubated (20 min–40 min, 37 °C, 120 rpm), and monitored with successive readings until the control absorbance had attained the range of 0.8 to 1.00 ± 0.1. A second reading was taken after incubation. Also, the absorbances were converted to % of inhibition. For the calculation of IC_50_, it was used Origin program by non-linear progression analysis.

*α-Amylase inhibition assay*: For α-amylase test, it was used an adapted methodology [22]. The reaction of substances diluted with the enzyme (α-amylase from human saliva, Sigma-Aldrich, A1031) was made on microplate. Then, 30 μL of inhibitor were added at the concentration of 1 mg/mL. After, it was incubated for 5 min (37 °C) and added 170 μL of substrate (CNPG Amylase Liquiform). The first reading ocurred at absorbance of 405 nm. The mixture was homogenized and incubated at 37 °C for 20–40 min until the control absorbance had reached 0.8 to 1.00 ± 0.1. The second reading was taken after incubation in microplate reader DTX-800 (Beckman Coulter, Inc., Brea, CA, USA) at absorbance of 405 nm. The results were expressed as % of inhibition and IC_50_ was calculated by the equation:

% Inhibition = 100 − (final abs − sample initial abs/final abs − initial abs of control) × 100.

## 4. Conclusions

In this study, a mixture of α,β-amyrenone was synthetized from α,β-amyrin, obtained from *Protium* sp. oleoresin. Its physico-chemical properties and in vitro biological activities were studied. The physico-chemical results allowed the identification and characterization of α,β-amyrenone. The XRD and SEM analyses, in addition to DSC, confirmed the crystalline nature of α,β-amyrenone, and corroborated the results of qualitative solubility that showed low aqueous solubility. α,β-Amyrenone, in comparison to α,β-amyrin, presents a smaller crystalline profile, less crystalline reflections, and better aqueous solubility. Selective inhibition of α-glucosidase and pancreatic lipase were observed in vitro. These results showed that this molecule has great potential for the development of new pharmaceutical forms and new drug delivery systems. Thus, the substance can be an elective choice for the production of formulations that may be used for the treatment of type II diabetes, metabolic syndrome and obesity disorders.
